# Regulation of Cardiac Remodeling by Cardiac Na^+^/K^+^-ATPase Isoforms

**DOI:** 10.3389/fphys.2016.00382

**Published:** 2016-09-09

**Authors:** Lijun Liu, Jian Wu, David J. Kennedy

**Affiliations:** ^1^Department of Medicine, College of Medicine and Life Sciences, University of ToledoToledo, OH, USA; ^2^Center for Craniofacial Molecular Biology, University of Southern CaliforniaLos Angeles, CA, USA

**Keywords:** cardiac remodeling, Na^+^/K^+^-ATPase, isoform, hypertrophy, fibrosis, cardiomyocyte, ouabain

## Abstract

Cardiac remodeling occurs after cardiac pressure/volume overload or myocardial injury during the development of heart failure and is a determinant of heart failure. Preventing or reversing remodeling is a goal of heart failure therapy. Human cardiomyocyte Na^+^/K^+^-ATPase has multiple α isoforms (1–3). The expression of the α subunit of the Na^+^/K^+^-ATPase is often altered in hypertrophic and failing hearts. The mechanisms are unclear. There are limited data from human cardiomyocytes. Abundant evidences from rodents show that Na^+^/K^+^-ATPase regulates cardiac contractility, cell signaling, hypertrophy and fibrosis. The α1 isoform of the Na^+^/K^+^-ATPase is the ubiquitous isoform and possesses both pumping and signaling functions. The α2 isoform of the Na^+^/K^+^-ATPase regulates intracellular Ca^2+^ signaling, contractility and pathological hypertrophy. The α3 isoform of the Na^+^/K^+^-ATPase may also be a target for cardiac hypertrophy. Restoration of cardiac Na^+^/K^+^-ATPase expression may be an effective approach for prevention of cardiac remodeling. In this article, we will overview: (1) the distribution and function of isoform specific Na^+^/K^+^-ATPase in the cardiomyocytes. (2) the role of cardiac Na^+^/K^+^-ATPase in the regulation of cell signaling, contractility, cardiac hypertrophy and fibrosis *in vitro* and *in vivo*. Selective targeting of cardiac Na^+^/K^+^-ATPase isoform may offer a new target for the prevention of cardiac remodeling.

## Introduction

Cardiovascular disease (CVD) is the leading cause of death in the United States, contributing to more than a third of deaths annually. Heart failure is a complex clinical syndrome resulting from structural or functional alterations in the heart which render it unable to meet the body's need for blood. In the United States, the risk of developing heart failure is 20% for the population aged 40 and over (Writing Committee et al., 2013). One in 9 deaths in 2009 included heart failure as contributing cause. Although the clinical management for heart failure has improved, mortality rates remain at ~50% within 5 years of diagnosis.

Cardiac remodeling occurs after cardiac pressure or volume overload or ischemic injury during the development of heart failure, and is a crucial factor in the prognosis of heart failure (Rizzello et al., 2009). Preventing or reversing remodeling is a goal of heart failure treatment (Konstam et al., 2011). Currently, beta-blockers and angiotensin converting enzyme (ACE) inhibitors or angiotensin receptor blockers (ARBs) are the first line of treatment in heart failure patients. Although several risk factors (hypertension, diabetes and coronary artery disease, etc.) have been identified, there are no effective strategies to prevent cardiac remodeling and heart failure.

Digoxin is the only FDA-approved cardiac glycoside. It can be beneficial in mild or moderate heart failure patients with reduced ejection fraction. Several clinical trials have shown that digoxin treatment improves symptoms and modestly reduces the combined risk of death and hospitalization (Writing Committee et al., 2013). Besides the known inotropy, effects of cardiac glycosides on cardiac remodeling have a long history. In 1933, Christian (1933) advocated the prophylactic use of digitalis to retard cardiac enlargement in heart disease patients without heart failure. In 1965, Williams and Braunwald (1965) presented the first experimental evidence that supports this proposal. Rats, subjected to suprarenal aortic constriction and treated with daily non-toxic doses of digitoxin prior to and following aortic constriction, exhibited less myocardial hypertrophy and a lower incidence rate of fatal heart failure than those subjected to aortic constriction but not treated with digitoxin. On the other hand, digoxin is reported to increase the mortality in chronic renal failure patients on dialysis (Chan et al., 2010). Infusion of cardiac glycosides causes reactive oxygen species stress (Charlemagne et al., 1994) and stimulates renal and cardiac fibrosis in animals with experimental renal injury (Kennedy et al., 2006; Elkareh et al., 2007; Fedorova et al., 2009).

The Na^+^/K^+^-ATPase is the only known receptor for cardiac glycosides (Shattock et al., 2015). It is unclear whether cardiac glycosides regulate cardiac remodeling and whether they can prevent or promote cardiac remodeling. The inconsistencies of the effect of cardiac remodeling may be due to the complexity of multiple isoforms of the α subunit and different binding properties of cardiac glycosides.

The Na^+^/K^+^-ATPase is an integrated membrane protein, which hydrolyzes ATP for the energy of the coupled active transport of Na^+^ and K^+^. It belongs to the P-type family of ATPase and consists of two non-covalently linked α and β subunits that are essential for ion pumping (Sweadner, 1989; Blanco and Mercer, 1998; Kaplan, 2002). The FXYD1 protein, i.e., phospholemman (PLM), is the third subunit in the heart that regulates the function of the enzyme (Geering, 2005). There are 3 isoforms of the α subunit (α1, α2, and α3) and 2 isoforms of the β subunits in the heart. The human heart expresses all 3 isoforms of the α subunit (Sweadner et al., 1994). Monkey and dog heart have roughly equal amount of α1 and either α2 or α3. Sheep and chicken heart only have the α1 isoform (Sweadner et al., 1994). The α1 isoform is ubiquitously present in the heart of all species. In the recent literature, a large amount of results came from rats and mice. In rodents, adult cardiomyocytes express mainly two isoforms of Na^+^/K^+^-ATPase α subunit, α1 (~75%) and α2 (≤25%), which have low and high affinities for ouabain, respectively (Sweadner et al., 1994; Bers et al., 2003; Verdonck et al., 2003).

In addition, different cardiac glycosides, e.g., ouabain and digoxin, have the same binding sites on the Na^+^/K^+^-ATPase although slightly different contacts may be made owing to the different sugar backbones (Laursen et al., 2015). Katz A. et al. have reported that glycosylation of cardiac glycosides contributes to human Na^+^/K^+^-ATPase isoform selectivity (Katz et al., 2010). At least in the case of digoxin, there is up to four-fold preference for α2 or α3 over the α1 isoform (Laursen et al., 2015). Those reports suggest that targeting different isoforms through modification of chemical structure of cardiac glycosides is a possible approach.

In this article, we will review the structural and enzymatic differences of α isoforms of the Na^+^/K^+^-ATPase in cardiomyocytes and the role of α isoforms of the Na^+^/K^+^-ATPase in cardiac hypertrophy and fibrosis.

## Enzymatic activity, distribution, and function of Na^+^/K^+^-ATPase isoforms in cardiomyocytes

The Na^+^/K^+^-ATPase (or sodium pump) was discovered in 1957 (Skou, 1957). It is a key enzyme in human cardiac myocytes (density up to 10^7^ molecules per cell) (Lelievr et al., 2001). This enzyme plays an important role in maintaining the cellular Na^+^ and K^+^ ion gradient, regulates cell volume, and enables the Na^+^-coupled transport of a multitude of nutrients and other ions across the cell membrane. Under normal conditions, the electrochemical potential gradient for Na^+^ ions, which the enzyme maintains, is one of the driving forces of Na^+^/Ca^2+^ exchanger to extrude intracellular Ca^2+^. In the classical ion pumping view of the Na^+^/K^+^-ATPase, when cardiac glycosides bind to the enzyme, they inhibit the active Na^+^ efflux and increase intracellular Ca^2+^ through Na^+^/Ca^2+^ exchanger. As a result, cardiac glycosides increase cardiac contractility.

The α subunit of the Na^+^/K^+^-ATPase is considered as the catalytic subunit and has ATP, cardiac glycosides, and other ligand binding sites. The β subunit is essential for the assembly of a functional enzyme (McDonough et al., 1990). There are multiple isoforms of each subunit with tissue and species specificities, and variations among the sensitivities of the isoforms to cardiac glycosides. In human cardiomyocytes, α1β1, α2β1, α3β1 are expressed in all regions (LA, RA, LV, RV, and S), while there is very low β2 expression in certain regions only (Wang et al., 1996; Schwinger et al., 2003). As judged by the sensitivities of Na^+^/K^+^-ATPase activity to ouabain (IC 50), the K_D_ values (measured by ^3^H ouabain binding), and by a biphasic ouabain dissociation process, at least two functionally active Na^+^/K^+^-ATPase isoforms coexist in normal human hearts (Lelievr et al., 2001): The IC 50 values are 7.0 ± 2.5 nM and 81 ± 11 nM; the K_D_ values in the presence of 10 mM [K^+^] are 17.6 ± 6 nM and 125 ± 25 nM; the dissociation rate constants are 360 × 10^−4^ min^−1^ and 42 × 10^−4^ min^−1^ (Lelievr et al., 2001).

There are observed kinetic differences (e.g., Km values for Na^+^, K^+^) among these isoforms, but their subtlety makes them an unlikely basis for physiological significance. Instead, recent work suggests that the major functional distinction among the isoforms is their interaction with regulatory proteins (Pressley et al., 2005). Isoform specific region among the isoforms are the NH2 terminus, the extracellular ouabain binding site, and the cytoplasmaic region between amino acids 403 and 503 (Blanco and Mercer, 1998). Moreover, the isoform-specific effects of modulatory proteins such as protein kinase C seem to originate within two regions of structural divergence: the amino terminus and the 11 residues near the center of the alpha subunit of the isoform-specific region (Blanco and Mercer, 1998; Pressley et al., 2005). The comparative protein model using human α sequencing based on pig α1 shows that there are very few isoform differences in the transmembrane and in the regions interacting with β and γ subunits; conversely, large clusters of isoform differences map at surface-exposed regions of the A- and N-domains (Morth et al., 2009). The structure difference may lead to isoform-specific cell signaling. Another example is that, in trafficking, α1 is recruited to the membrane by adaptor protein 1 via Tyr 255, a residue not conserved in other isoforms (Cinelli et al., 2008).

**Figure 1:**
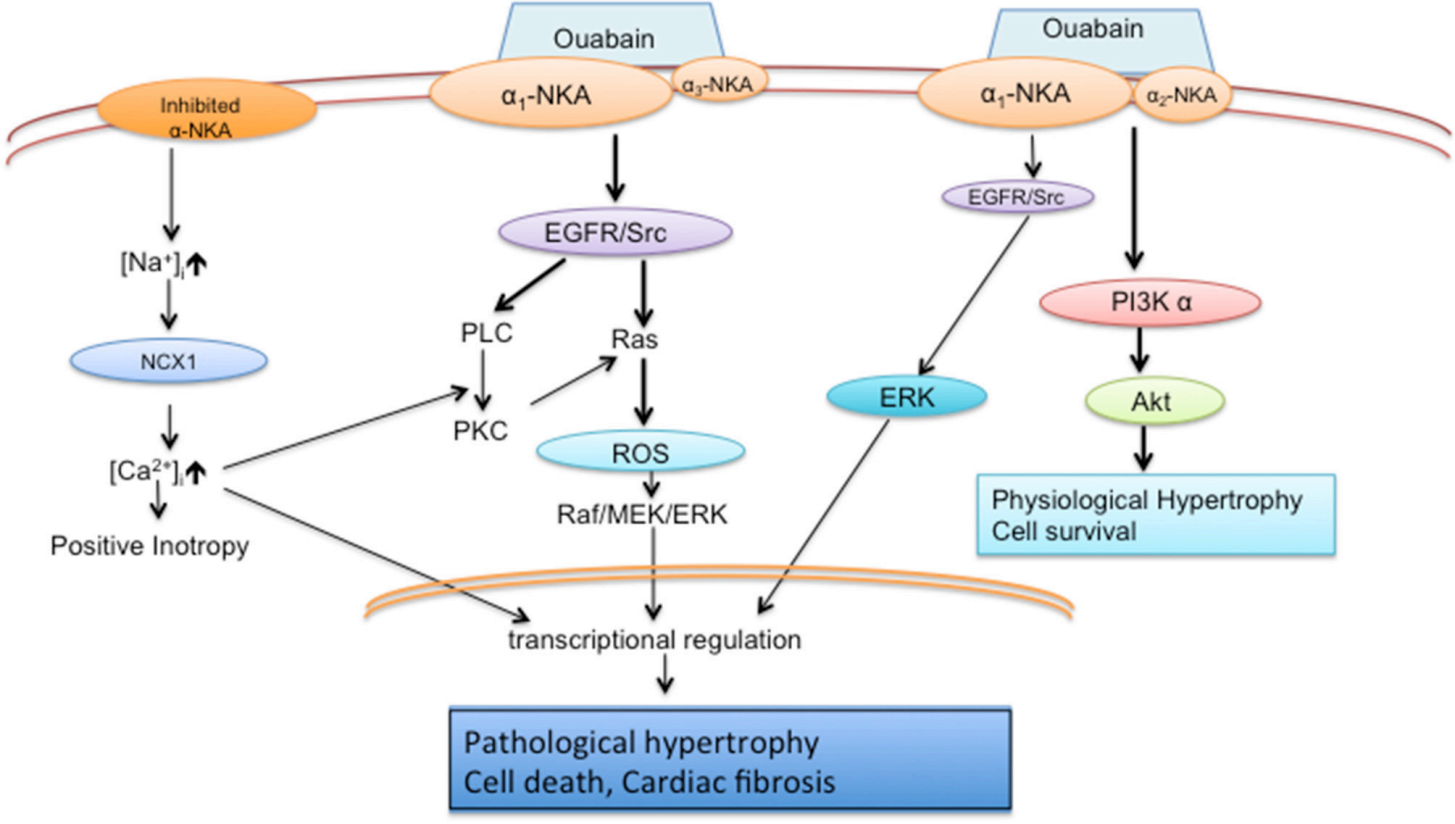
**Schematic diagram of Na^**+**^/K^**+**^-ATPase pumping and signaling functions in cardiomyocytes (Liu et al., 2006, 2007; Wu et al., 2015)**. Inhibited pump alters local [Na^+^]_i_ and induces myocytes contractility; Major effect of ouabain signaling is Src/Ras/ROS/ERK cascade in α1/ α3 neonatal cardiomyocytes; Major effect of ouabain signaling is PI3Kα /Akt pathway in α1/ α2 adult cardiomyocytes.

Besides its ion pumping function, the Na^+^/K^+^-ATPase also serves as a scaffold protein interacting with neighboring proteins and facilitates multiple cell signaling events. As shown in Figure 1, Na^+^/K^+^-ATPase signaling mainly has two parallel pathways, one is the EGFR/Src/ERK pathway and another is the phosphoinositide 3-kinase (PI3K) α/Akt/β-GSK/mTOR pathway. To be sure there is some debate regarding the nature of the interaction between the Na^+^/K^+^-ATPase and Src (Weigand et al., 2012; Clifford and Kaplan, 2013; Yosef et al., 2016). Ouabain indeed does activate Src in myocytes (Haas et al., 2000; Mohammadi et al., 2001, 2003; Xie and Askari, 2002; Liu et al., 2003, 2004) and several lines of evidence support the finding that the Na^+^/K^+^-ATPase and Src do interact and induced by ouabain or high salt by immunoprecipitation assay in cardiomyocytes, breast cancer cells, and primary pig proximal tubular cells and LLC-PK1 cells (Mohammadi et al., 2003; Kometiani et al., 2005; Liu et al., 2011; Yan et al., 2013). However, the mechanisms of the interaction between the Na^+^/K^+^-ATPase and Src are unclear. The mechanistic analysis on living cells are required to clarify the complicated network in cells. Thus, some of the conflicting results regarding the interaction between the Na^+^/K^+^-ATPase and Src may be related not only to cell specificity and specific *in vitro* conditions with detergent-treated membrane purified sodium pump.

The α1 and α2 isoforms play different roles in cardiomyocyte function. There is ample evidence of α1 isoform signaling (Xie and Askari, 2002; Bossuyt et al., 2009; Han et al., 2009; Shattock et al., 2015; Stanley et al., 2015) while no direct evidence is shown on the α2 isoform signaling in cardiomyocytes. Based on different affinities of α1 and α2 for ouabain in mice, and the cardiomyocytes detubulation, Berry et al. (2007) found that α1 is the predominant current conductor, contributing 88% of total recordable current *I*_total−pump_. Although *I*_α1_ density predominates over *I*_α2_ in both the surface sarcolemmal membrane (SSL) and T-tubules, the difference of current density between α2 and α1 is markedly decreased in the T-tubule. It was reported that α1 is uniformly distributed between SSL and T-tubules, while α2 is ~5 times more concentrated in T-tubules; strongly suggesting a different role of two isoforms in cardiomyocytes. A similar distribution pattern of two isoforms was recapitulated in rat cardiomyocytes (Despa and Bers, 2007; Swift et al., 2007). NCX and L-type Ca^2+^ channels are also enriched in T-tubules. Both α1 and α2 were shown to be functionally and physically coupled with NCX in cardiomyocytes; however, they differ in their effects on intracellular Ca^2+^ through regulating [Na]_i_ (Yamamoto et al., 2005). The α1 isoform regulates global [Na]_i_, while α2 controls local [Na]_i_. α2 isoform is reported to regulate calcium (James et al., 1999), preferentially modulate Ca^2+^ transients and sarcoplasmic reticulum Ca^2+^ (Despa et al., 2012). Recent reports have shown that the α2 isoform of the Na^+^/K^+^-ATPase is inactive during the resting potential in adult rat cardiomyocytes and primarily affects calcium handling during systole (Stanley et al., 2015). This suggests that the α2 isoform of Na^+^/K^+^-ATPase is a specific voltage-dependent isoform (Stanley et al., 2015).

Adrenergic signaling may affect Na^+^/K^+^-ATPase activity. This effect is stimulated by PKA and/or PKC and phosphorylation of FXYD1 (phospholemman, PLM) in hearts (Bers and Despa, 2009). Cardiac ischemia induces PKA-dependent phosphorylation of PLM and activates α1 Na^+^/K^+^-ATPase activity but not α2 in rats (Fuller et al., 2004). Interestingly, α-adrenergic agonist increases α2 specific Na^+^/K^+^-ATPase activity in guinea-pig cardiomyocytes (Gao et al., 1999). Bers and colleagues clearly showed that β-adrenergic signaling stimulates α1 Na^+^/K^+^-ATPase activity, but not α2 activity on mouse cardiomyocytes (Bossuyt et al., 2009). Similarly, forskolin (activating cAMP/PKA, the downstream of β-adrenergic signaling) also specifically activates α1 Na^+^/K^+^-ATPase activity in guinea-pig cardiomyocytes (Gao et al., 1999; Silverman et al., 2005). Furthermore, the combination of β- and α -adrenergic signaling in the heart somehow leads to dramatic reduction of α2 but not α1 (Sjogren et al., 2016). While these effects of β-adrenergic stimulation have been documented in rodent cardiac myocytes, it is less clear how this may translate to human cardiac myocytes especially in the setting of heart failure where reductions in α1 and α2 Na^+^/K^+^-ATPase have been noted.

## Alterations of Na^+^/K^+^-ATPase isoforms in cardiac hypertrophy

Isoforms of the cardiac Na^+^/K^+^-ATPase play different roles in cardiac hypertrophy (Huang et al., 1997a; Kometiani et al., 1998; Xie and Askari, 2002; Bai et al., 2013). Most data shown in the literature are in rodent. While there are α1 and α2 isoforms in adult cardiomyocytes, α1 and α3 isoforms are expressed in neonatal cardiomyocytes.

In cultured neonatal cardiomyocytes, hypertrophic stimuli that mimic pressure overload induces reduced Na^+^/K^+^-ATPase activity and the regression of α3 mRNA and protein without the alteration of α1 mRNA and protein (Huang et al., 1997b).

Ouabain activates phosphoinositide 3-kinase (PI3K) α /Akt/β-GSK/mTOR and lead to physiological hypertrophy in cultured adult cardiomyocytes (Liu et al., 2007; Bai et al., 2013; Wu et al., 2015). It is featured different from pathological hypertrophy as no increase the influx of Ca^2+^ (Bai et al., 2013) and hypertrophic markers (ANP and BNP) in hypertrophic myocytes (Bai et al., 2013; Wu et al., 2015).

Regression of the ouabain-sensitive isoform α2 is the marker associated with cardiac hypertrophy *in vivo* (Book et al., 1994; Charlemagne et al., 1994; Wu et al., 2015), although several reports have demonstrated that the ouabain-resistant isoform α1 is also downregulated in cardiac remodeling (Norgaard et al., 1988; Semb et al., 1998; Borlak and Thum, 2003; Zwadlo and Borlak, 2005; Kennedy et al., 2006). The α2 isoform mRNA and protein are decreased during hypertrophy of the left ventricle, e.g., in pressure-overload (Book et al., 1994; Ruiz-Opazo et al., 1997; Rindler et al., 2013), isoprenaline-induced cardiac hypertrophy (Baek and Weiss, 2005), myocardial infarction (Book et al., 1994), and uremic cardiomyopathy (Kennedy et al., 2006). Alteration of the α2 isoform of the Na^+^/K^+^-ATPase may be a mechanism for pressure overload-induced transcriptional response (Ruiz-Opazo et al., 1997). This downregulation of the α2 isoform attenuates the control of Na^+^/Ca^2+^ exchanger (NCX) activity and reduces the capability to extrude Ca^2+^ from cardiomyocytes (Swift et al., 2008). In failing hearts, the α2 isoform are correlated to increases Ca^2+^ cycling (Liu and O'Rourke, 2008) and disorganized T-tubule network in cardiomyocytes (Swift et al., 2008). However, the cause-and-consequence of down-regulation of α2 in cardiac remodeling is unclear.

It is interested to know if the compensation between the isoforms and interaction among the isoforms and other proteins would be true in human heart. In α1^+∕−^ heterozygote mice, cardiac α2 was increased 50%. In α2^+∕−^ heterozygous mice, α1 was not changed but NCX was dramatically increased (Yamamoto et al., 2005). Another example is Ankyrin-B. Ankyrin-B is a universal cell membrane adaptor protein. It may be the scaffold protein for the interaction between Na^+^/K^+^-ATPase and NCX. Reduced T-tubular α1 and α2 were shown in the mice with heterozygous knockdown of Ankyrin-B (Mohler et al., 2003).

Overexpression of cardiac-specific α2 but not α1 (Correll et al., 2014) protects the heart from pressure overload induced cardiac hypertrophy, fibrosis, and cardiac dysfunction, suggesting that α2 regulates cardiac pathological hypertrophy. Na^+^/K^+^-ATPase α2 overexpression does not block TAC-induced pro-hypertrophic signaling pathways, such as previously established Ca^2+^/calmodulin-dependent protein kinase II (CaMKII) and nuclear factor of activated T cells (NFAT) (Correll et al., 2014), but its impact on NCX1 is sufficient to improve cardiac function during the cardiac remodeling. The possible mechanisms may be because overexpression of α2 decreases PLM levels and phosphorylation. PLM is an inhibitor of Na^+^/K^+^-ATPase activity. Although both α1 and α2 isoforms directly couple to NCX1, α2 isoform is much more enriched in T-tubules and partial inhibition of α2 but not α1 can increase Ca^2+^ transients suggesting α2 isoform is responsible for regulating NCX1 to control intracellular Ca^2+^. [Na^+^] curve of Na^+^/K^+^-ATPase activity in overexpressed α2 myocytes shifted to left compared to control and α1 overexpression suggesting α2 has a higher affinity and it may be due to less regulated by PLM. Intracellular Na^+^ plays a crucial role in contractility and cardiac remodeling in failing hearts because lower intracellular Na^+^ results in less damage to mitochondria and reduction in ATP production (Pieske et al., 2002; Pogwizd et al., 2003).

Cardiac-specific α2 isoform knockout mice are able to survive and have no change in baseline cardiac function (Correll et al., 2014). Reversing the sensitivities of the α1 and α2 isoform to ouabain causes more severe hypertrophy and fibrosis by pressure-overload (Wansapura et al., 2011). These findings indicate that α1 and α2 isoforms play distinct roles in regulating cardiac remodeling.

To be sure the relative insensitivity of rodent Na^+^/K^+^-ATPase compared to human is well known and is an important methodological limitation in studies that examine Na^+^/K^+^-ATPase interactions with cardiac glycosides, such as those referenced above. Thus, these studies need to be interpreted in light of this important limitation and care needs to be given to extrapolating their relevance to humans, especially as these differences may be exacerbated in the heart.

## Ouabain infusion in cardiac remodeling

Infusion of ouabain (15 μg/kg/day × 18 weeks) doubles plasma “ouabain-like” immune-reactive material from 0.3 to 0.7 nM and induces hypertension as well as cardiac and renal hypertrophy in rats (Ferrandi et al., 2004). Other studies (Manunta et al., 1994, 2000; Huang and Leenen, 1999; Rossoni et al., 2002) also showed that infusion of ouabain (25–30 μg/kg/day) for 5 weeks induces hypertension in rats. Rats are much more sensitive to hypertension compared to mice via ouabain. However, other researchers stated that cardiac hypertrophy in rats by ouabain is independent of hypertension (Jiang et al., 2007).

Conversely, ouabain does not induce cardiac hypertrophy in mice; moreover, ouabain is protective against pathological hypertrophy. Infusion of ouabain (300 μg/kg/day) induces hypertension in mice and results in 3.3 nM of “ouabain-like” immune-reactive material (Dostanic et al., 2005). Infusion of ouabain (50 μg/kg/day × 4 weeks) does not induce mouse hypertension and cardiac hypertrophy *in vivo* (Wu et al., 2015). Others (Dostanic et al., 2005) also reported that the repeated daily administration of 100 μg/kg of ouabain to mice resulted in no significant change (the range of 0.75–0.87 nM) in serum of “ouabain-like” immune-reactive material and no effect on systolic blood pressure. The minimal dose of ouabain causing positive inotropic effect is noted to be 40 nM in isolated perfused heart (Dostanic et al., 2003). Infusion of ouabain (50 μg/kg/day) with an implantable osmotic pump in mice for the first 4 weeks starting 1 day after transverse aortic constriction (TAC) prevents pressure-overload-induced cardiac hypertrophy (Wu et al., 2015). The prophylactic effect of sub-inotropic and sub-nanomolar dose of ouabain is associated with activation of PI3Kα (Wu et al., 2015). Ouabain also attenuates TAC-induced reduction of the α2 Na^+^/K^+^-ATPase. These results demonstrate the regression of α2 Na^+^/K^+^-ATPase in cardiac hypertrophy and suggest that preservation of the α2 Na^+^/K^+^-ATPase improves cardiac function and prevents cardiac hypertrophy. These data provide experimental evidence that ouabain can be beneficial to stage A [at high risk for heart failure (HF) but without structural heart disease or symptoms of HF] and B (structural heart disease but without signs or symptoms of HF) patients but not the stage C (structural heart disease with prior or current symptoms of HF) and D (refractory HF requiring specialized interventions) patients [according to the American Heart Association (AHA) and American College Cardiology Foundation (ACCF) guideline (Writing Committee et al., 2013)].

Intriguingly, recent findings by Neubig and colleagues (Sjogren et al., 2012, 2016) have shown that by screening several thousand compounds, digitalis drugs (including ouabain and digoxin) are able to stabilize RGS2 protein, a molecular brake for overdriven Gq signaling in the diseased heart. More interestingly, they further proved the stabilization of RGS2 protein by very low concentration of digoxin (2 μg/kg/day, 7 days) protects heart from injury in mice (Sjogren et al., 2016). However, it is unclear that the impact of digitalis drugs on RGS is direct or indirect via Na^+^/K^+^-ATPase.

## Oxidative stress, endogenous cardiotonic steroids, and cardiac fibrosis

Ouabain activates membrane receptor tyrosine kinase and Src/Ras and results in increase of mitochondrial reactive oxidase species (ROS) in cardiomyocytes (Liu et al., 2000, 2006). Ouabain-induced ROS is independent of the changes of intracellular calcium and sodium (Xie et al., 1999; Liu et al., 2000, 2006). ROS are not contributed to the positive inotropic effect of ouabain, but a ROS-dependent pathway is involved in ouabain-induced hypertrophy (Xie et al., 1999), and contributes to gene transcriptional regulation of hypertrophy (Liu et al., 2000; Liu and Xie, 2010).

Since “endogenous ouabain” in human was first reported in 1991 (Hamlyn et al., 1991), many research groups have isolated Na^+^/K^+^-ATPase ligands and identified them as “ouabain-,” “digoxin-,” “marinobufagenin (MBG)-,” and “telocinobufagin”-like steroids (Hamlyn, 2014) and collectively referred to as “cardiotonic steroids” (Bagrov et al., 2009). Several clinical and experimental studies have reported that endogenous Na^+^/K^+^-ATPase ligands act as natriuretic hormones and are elevated in cardiovascular and renal diseases (Kolmakova et al., 2011; Kennedy et al., 2015). “Endogenous ouabain” in the immediate postoperative period was strongly indicative of a more severe cardiac disease and predicts mortality in heart failure patients undergoing elective cardiac surgery (Simonini et al., 2015). There are debates and inconsistencies within this field. For example, conversely, other research groups were not able to detect plasma endogenous ouabain in any conditions (Baecher et al., 2014; Lewis et al., 2014). Technical problems on the measurement of these “endogenous” steroids need to be resolved in the near future.

The hypothesis proposed by Blaustein (2014) is that endogenous ouabain (EO) has similar short term effect but different long term effect than that of digoxin. Long-term effects of EO cause hypertension and heart failure (Blaustein, 2014). Fedorova and coworkers demonstrated that in response to salt loading, transient increases in brain EO, stimulates adrenocortical MBG via an angiotensin I receptor pathway resulting in renal sodium pump inhibition and elevation in blood pressure (Fedorova et al., 2005). Nevertheless, Endogenous MBG affects different signaling pathways and functions from ouabain in the heart. Plasma MBG is elevated in uremic cardiomyopathy in rats (Haller et al., 2012), as well as in the left anterior descending (LAD) ligation model of heart failure in mice (Fedorova et al., 2015a; Kennedy et al., 2015). Elevated plasma MBG is correlated with higher blood pressure, especially in salt sensitive men (Fedorova et al., 2015b). Moreover, MBG stimulates collagen synthesis and cardiac fibrosis (Elkareh et al., 2007; Kennedy et al., 2015; Drummond et al., 2016). Infusion of MBG to mice increases nitrative stress and cardiac fibrosis (Fedorova et al., 2015a; Kennedy et al., 2015), while monoclonal antibodies against MBG are able to reverse cardiac fibrosis in uremic cardiomyopathy (Haller et al., 2012). MBG (100 nM) also resulted in a two-fold rise in collagen-1 in cultured rat aortic smooth muscle cells and a marked reduction in the vasorelaxation following endothelin-1 stimulated constriction in the aortic rings (Fedorova et al., 2015a). Elevated MBG levels are associated with worsened right ventricular function even after controlling for age, sex, diabetes mellitus, and ischemic pathogenesis in humans (Drummond et al., 2016). Clearly, MBG exhibits at least some of its effects via the α1 isoform as infusion of MBG causes cardiomyocyte death and dilated cardiomyopathy in α1 knockdown mice (Liu et al., 2012).

## Summary

The Na^+^/K^+^-ATPase α isoforms play an important role in the regulation of cardiac remodeling. A schematic diagram that visually summarizes the reviewed results and conclusion is presented to assist readers in efficiently seeing the proposed interaction effects in Figure 1. The α1 isoform regulates cell growth and survival pathways, ROS generation, hypertrophy, and cardiac fibrosis. While the α2 isoform has known functions in the regulation of calcium, contractility and hypertrophy on cardiomyocytes, further work will be necessary to delineate any signal transduction role beyond these known functions. While human cardiomyocytes contain three α isoforms, much work remains to determine their function in the healthy and diseased heart and their potential contribution to cardiac remodeling. The Na^+^/K^+^-ATPase α isoforms are positioned as promising therapeutic targets that can be exploited in both the prevention and treatment of heart failure. As such, future mechanistic work investigating the contribution of specific isoforms of the Na^+^/K^+^-ATPase will not only advance our understanding of cardiac remodeling but may also provide insight into novel treatment strategies for patients with heart failure.

## Author contributions

LL designed, wrote and revised the article; JW wrote the article; DK wrote and revised the article.

## Funding

This study was supported by NIH grant PO1 HL036573 and University of Toledo, College of Medicine and Life Sciences bridge fund (LL). American Heart Association Scientist Development Grant (12SDG12050473, DK), the David and Helen Boone Foundation Research Fund (DK), and an Early Career Development Award from the Central Society for Clinical and Translational Research (DK).

### Conflict of interest statement

The authors declare that the research was conducted in the absence of any commercial or financial relationships that could be construed as a potential conflict of interest.
